# Building a Local Research Symposium: The Crossroads of Scholarship, Education, and Faculty Development

**DOI:** 10.15766/mep_2374-8265.11048

**Published:** 2020-12-24

**Authors:** Adam D. Wolfe, Utpal Bhalala, Vivienne Marshall

**Affiliations:** 1 Assistant Professor of Pediatrics, Associate Residency Program Director, Director of Faculty Development, and Assistant Dean of Education, Baylor College of Medicine at The Children's Hospital of San Antonio; 2 Assistant Professor of Pediatrics, Director of Pediatric Critical Care Research, and Medical Director, Voelcker Clinical Research Center; Founding Chair of The Children's Hospital of San Antonio Research Steering Committee, Baylor College of Medicine at The Children's Hospital of San Antonio; 3 SW USA Research and Development Manager, Canopy Growth Corporation

**Keywords:** Scholarship, Research Symposium, Community-Based, Communication Skills, Faculty Development, Publishing/Scholarship, Self-Regulated Learning, Editor's Choice

## Abstract

**Introduction:**

Demonstrating research productivity for faculty and trainees is challenging in primarily community-based settings, where academic, structural, and financial resources for faculty development in scholarship may be limited. More tools are needed to guide faculty leaders in community-based settings to develop opportunities locally.

**Methods:**

At our community-based children's hospital with recent academic affiliation and a new residency program, we developed an annual research symposium targeted to faculty and trainees. We refined tools for solicitation and scoring of abstracts, speaker selection, skill-building workshops, scholarly case report presentations, and a mentored poster session. We worked with available resources, kept costs flexible and low, and secured local partnerships to defray expenses. Evaluation consisted of session evaluations and trends in abstract submissions, institutional review board (IRB) submissions, and resident scholarly productivity over 4 years.

**Results:**

Scholarship improved over the symposium's first 4 years, with increased attendance (from 80 to 150), abstract submissions (from 29 to >50), IRB-approved research projects (from 65 to 123), and positive feedback on symposium evaluations. From our first three resident classes, 61 resident-authored abstracts were presented at our symposia, with 33 presented at regional and national meetings and 15 converted to peer-reviewed manuscripts.

**Discussion:**

We have developed a local research symposium to meet the needs of a new hospital's faculty and trainees. Evaluation data have allowed us to tailor the program to stakeholder needs. We provide a tool kit of generalizable resources for community-based programs to build on these efforts in a high-yield and cost-effective manner.

## Educational Objectives

By the end of this activity, participants will be able to:

1. Participate in a research symposium designed to enhance faculty and trainee scholarship.
2. Submit a scholarly abstract for a local symposium.
3. Practice presenting a scholarly project as a poster or oral presentation in a faculty-facilitated setting.
4. Describe clinical teaching cases that merit publication.

## Introduction

Accomplishing scholarly activity is recognized as a challenge for many clinical faculty members^1^ and trainees,^2^ particularly in primarily clinical or community-based settings.^3^ For leaders of GME programs, scholarship of learners (i.e., residents and fellows) and faculty is a requirement for continued program accreditation.^4^ Over time, the Accreditation Council for Graduate Medical Education (ACGME) has revised guidelines for scholarship to increase the emphasis on scholarship as a cornerstone of educational program success.^5^

Community-based GME programs, with or without university affiliations, are numerous and crucial for training adequate numbers of clinicians to meet demand.^6^ The teaching faculties at these programs are expected to demonstrate evidence of scholarship for their own professional development^3^ and for support of learners.^4,5^ This may pose a challenge for educational and faculty development leaders, depending on the setting. For example, a survey of directors of pediatric residency programs identified the participants' top two concerns to be (1) promoting faculty scholarly activity and (2) faculty development.^7^

Resources for improving trainee scholarship currently available in *MedEdPORTAL* include training curricula^8–10^ and tools to encourage and teach faculty mentorship of learners.^11,12^ Strategies for improving faculty scholarship include skill-building^1,2^ and incentive-based^13^ interventions.

While many large academic centers offer established local or regional research meetings, there is little guidance in the literature as to how to put this into place in a smaller or community-based setting. *MedEdPORTAL* does not currently include a guide for leaders to develop, fund, and conduct a local research symposium, although some of the scholarly project resources include to-do lists of skills needed to successfully conduct research.^14^ The *MedEdPublish* database offers several resources in project management skills, which may prove helpful in organizing an event of the complexity of a symposium,^15,16^ and several brief tip lists for running meetings are available on the internet.^17–20^

As a joint effort of our leaders in research, GME, and faculty development, we have created a local symposium to serve as an annual opportunity for the entire hospital community to share findings, collaborate on future projects, and build new skills in scholarship. The symposium has been refined over four annual cycles of needs assessment, program development, implementation, and evaluation to continue to meet the needs of learners, faculty, and hospital associates.

This resource provides the tools necessary to plan a local research symposium in a community-based clinical setting. We include materials for needs assessment, planning, publicity, abstract management, scholarly and educational content, budgeting, and evaluation. This event has been increasingly successful each year, as measured by evaluation data, number and quality of abstracts submitted, number of research studies opened at our institution, scholarly productivity of residents, and continued expansion of the program. Based on our first 4 years of experience, we offer suggestions for ways to customize the symposium based on an institution's strengths, resources, and needs. While this resource was developed in a children's hospital with faculty and learners in pediatrics, we believe the materials are not pediatrics specific and should be equally appropriate to adapt for any community-based setting or academic health center.

## Methods

### Program Planning

Annual symposium planning began with an online needs assessment (Appendix A). The responses allowed identification of symposium content (e.g., workshops, grand rounds topics) that could best meet needs identified by faculty.

Guided by the identified needs, the program consisted of an invited grand rounds speaker, oral abstract presentations, faculty-moderated poster session, clinical case presentations, and workshops (Appendix B). We developed a chronological checklist of tasks for our symposium organizers to complete throughout the planning of the event (Appendix C).

### Abstract Submission and Review

We solicited abstracts from colleagues throughout the hospital, including faculty, residents, fellows, students, pharmacists, nurses, dietitians, and child life specialists, as well as from colleagues at other programs in our city that conduct clinical research relevant to pediatrics. The call for abstracts was opened in early summer, with a deadline approximately 1 month before the September symposium. We solicited abstracts using a free Google Forms account (Appendix D). This approach allowed for organization of submissions into a simple spreadsheet that was used for both abstract review tracking and subsequent communication with authors.

Following the abstract submission deadline, our leadership team met to review abstracts. For development of the process, we felt that the abstract reviewers should have broad experience in research and education. Our initial review team consisted of three faculty members who represented expertise in research process and methodology, scholarly project design, technical writing, research regulatory process, and medical education. All three had prior experience with reviewing abstracts for regional or national meetings. Together, we developed an abstract scoring rubric that was brief and criterion based. The rubric was implemented and refined over 4 years of symposia by the same group of three faculty leaders at our institution (Appendix E). We planned this process such that future symposium leaders would not need substantial prior experience to conduct this review, using a rubric sufficiently simple as to apply to abstracts from multiple domains of scholarship.

Abstracts were clustered into scholarly domains of basic science research, translational research, clinical research, quality improvement/patient safety, education, advocacy, community health/global health/epidemiology, and clinical conundrum/case report. The clinical conundrum/case report category was treated separately (see below), and all other categories were assigned a review score out of 18 by each reviewer (Appendix E). Following independent review and scoring, the reviewers met to compare scores and to identify top-scoring abstracts to invite for oral platform presentations. Since we had a goal of showcasing and promoting different scholarly project domains, we prioritized inviting at least one research, one quality improvement/patient safety, one education, and one advocacy abstract for platform presentation when possible. Oral presentation selection was completed during a 1-hour meeting. All abstracts not selected for oral presentation were accepted for poster presentation; in order to allow authors to receive feedback on their work during the moderated poster session, no abstracts were rejected.

Communication with abstract authors was conducted using the data from the submission form (Appendix D). Using the mailings feature in our word-processing software, we imported the spreadsheet data from the submission form and customized bulk emails that were distributed to the authors regarding oral and poster acceptances (Appendix F).

### Poster Session Preparation

Based on previous experience with poster-printing costs of up to $7–$20 per square foot, we called locally operated print shops throughout the city and found a shop manager who was willing to offer a 20% discount on poster printing, which reduced the average cost for printing a matte-finish, 4×4 poster from $120 to $96. We also encouraged posters that had been presented during the past year at national or regional meetings to be presented at this local event without reprinting, to increase the audience for (and pride in) local institutional research.

Our venue of several adjacent classrooms provided ample wall space for hanging posters within the rooms and on hallway walls between rooms. We identified two poster-hanging strategies that were equally successful. We hung posters on smooth or textured flat surfaces using poster putty, a removable adhesive material available from multiple manufacturers; a 2-ounce package proved sufficient to hang approximately 10–12 posters. We also used Command brand adhesive poster strips (3M). These were removable, had a broader surface area of adhesion, and proved less likely to separate from the wall over time. We hung posters on classroom walls, window shades, and the walls in the hallway between rooms. Neither of our mounting methods resulted in any damage to painted, enameled, fabric (Command strips only), or wood surfaces on which we hung posters.

We reserved our poster session space to include 1 day prior to the symposium. Our program leaders measured out 5-foot spaces along the walls and placed small number cards in each space using poster putty. Our presenters were invited to hang their posters 1 day early if this fit well with their schedules (Appendix G). We also opened the space early in the morning on the day of the symposium for poster hanging. Each poster was placed at its assigned number based on groupings for the facilitation process (see below).

### Poster Session Facilitation

We conducted professor walk rounds during our poster session. This mentored session involved recruiting two faculty members per six posters being presented to serve as moderators/judges. Faculty members selected for this process did not require any special experience, and training consisted of receiving written instructions to review in advance (Appendices G and H).

Each group's posters were hung adjacent to each other by assigned numbers. At the start of the author-attended poster session, all of the authors for each small group met with their faculty moderators/judges and systematically rounded on each poster. Each presenter had 5 minutes to present the poster, followed by 5 minutes of questions and critique by group members. Faculty moderators/judges scored each poster using a rubric we modified from that used by the Society for Critical Care Medicine (SCCM) and refined over 3 years of implementation (SCCM rubric supplied to conference abstract reviewers and not publicly available; adapted as shown in Appendix H).

At the conclusion of the poster session, we presented awards for the top-scoring faculty-authored poster presentation and the top-scoring trainee-authored poster presentation. These were given in the form of a $100 gift card each.

### Oral Platform Sessions

The most favorably reviewed abstracts were invited for oral platform presentations (see above). The space requirement for this session was a room sufficiently sized to seat all participants and with a computer and central projection screen for slides. We used our hospital's auditorium, which seated approximately 100, for this purpose. Each presenter was allowed 15 minutes for each presentation and 5 minutes for questions. We offered seven platform presentations separated into two sessions (Appendix B). In order to keep the transitions between speakers to a minimum, we asked the session presenters to send in their electronic slides ahead of time. These were then loaded onto the computer used for the presentations at the beginning of the day.

### Clinical Conundrums: “2 Minutes, 2 Slides, 2 Questions” Format

Many trainee and faculty abstracts described case reports or small case series. While our scoring rubric was not designed for case reports, we wanted to offer an opportunity for the authors to hone their presentation skills and to recognize the value that a scholarly approach to a teaching case could have in informing their future scholarly pursuits.

In addition to presenting their cases as posters, we invited case report authors to participate in a separate oral presentation session called “2 Minutes, 2 Slides, 2 Questions: Clinical Case Symposium.” Held during an hour of the symposium day, this session was adapted from a format incorporated at other meetings (D. Bernhardt, MD, email, September 10, 2016).^21^ Each presenter received 2 minutes for an oral presentation about the case and a single teaching point. The presentations were timed and tightly moderated, and each speaker concluded when the timer expired. Speakers were allowed to present information from up to two electronic slides during their 2 minutes and then could address up to two audience questions. We budgeted 4 total minutes per presentation to incorporate time for questions and transition to the next speaker, allowing up to 15 presentations to be scheduled in a single hour (Appendix B).

All speakers were required to submit their slides in advance. These were assembled into a single slide deck to further compress the time required for the session.

### Workshop Selection and Facilitation

We offered four scholarly skill-building workshops (topics we have offered are shown in Appendix B). Workshops were offered as 90-minute interactive sessions. The agenda included two concurrent workshops in the morning and two additional concurrent workshops in the afternoon. Workshop proposals were solicited by email from all faculty 3–4 months before each symposium, and certain faculty members were also specifically invited to submit proposals based on their known expertise. Annual workshop topic selection has been guided by needs assessment (Appendix A) and evaluation data from previous years.

Each workshop was presented in a small classroom set with round tables, with seating capacity of at least 20 participants. Each room had a projection screen and computer. Physical handouts were supplied by workshop facilitators as needed.

### Grand Rounds (Keynote) Speaker

We partnered with the hospital Grand Rounds Planning Committee to include the morning grand rounds session as part of our symposium day. Speaker selection was guided by our needs assessment. Past selections have focused on how individual clinical experiences can stimulate a career in academic medicine and on how networking and collaboration can help one develop robust research productivity. Our grand rounds series was offered with continuing medical education credit, which we expected would facilitate greater attendance for the start of each symposium.

### Printed Materials

Beginning in the second year, we prepared a printed symposium program including the day's agenda as well as workshop, oral, and poster abstracts being presented throughout the day. Inclusion of the program was intended to assist participants in planning their day and to provide all of our presenters with written dissemination of their abstracts. Several word-processing or publishing software products proved capable of creating the program layout. We printed the program on our office printer to avoid incurring the costs of professional printing. We developed a list of print materials to prepare ahead of the research symposium (Appendix C).

### Budgeting and Funding

Recognizing that, in a community-based setting, we would have limited opportunities for ambitious funding, we intentionally kept our costs as low as possible by using free online resources, available software, available space and physical resources, and personnel already dedicated to the project. To cover necessary expenses, we created partnerships with several different groups within the hospital. Funds were contributed by the Grand Rounds Committee, the GME office, the faculty development budget, the hospital's Clinical Research Center, The Children's Hospital of San Antonio Foundation, and our department chair's discretionary funds. All communications and print materials were prepared by a member of our planning committee and incurred no additional expense. Poster-printing costs were covered by presenters, some of whom had section chief discretionary funds to assist them. A sample budget is included in Appendix I.

### Program Evaluation

For participant evaluation of the symposium, we utilized paper evaluation forms. Each session of the symposium (oral sessions, clinical case symposium, poster session, each workshop) had a separate paper evaluation printed on a distinct color of paper (Appendix J). We provided evaluations directly to the participants during each session. Evaluation return boxes were placed in every room of the symposium sessions, and participants were encouraged to deposit their evaluations in real time at the conclusion of each session. Following the symposium, the evaluations were sorted by color (i.e., by session), scanned, and distributed to the appropriate leaders and presenters to guide improvement of their work. The symposium planning committee reviewed all evaluations to determine which sessions were most successful and which topics the participants identified as needs for the following year.

Data analysis of symposium evaluations was conducted by our three symposium codirectors. We calculated the average scores of the Likert-response questions, with particular attention to the question “I will apply what I learned during this session” as an assessment of participants' intention to practice new knowledge or skills (Appendix J). Narrative responses to the open-ended questions on the evaluation forms were transcribed verbatim into a typed word-processing document. Comments were grouped into those that identified strengths and areas for improvement in the program. Shared themes were then identified, and the number of comments relating to each theme were tallied. All themes with two or more instances within the comments are reported (see Results, below).

Additional quality measures were selected to assess the change in overall scholarly activity at our institution over the first 4 years of the symposium. We observed the trends in numbers of abstracts submitted to the symposium year over year. Additionally, we tracked the number of institutional review board (IRB) submissions for research studies at our institution prior to the first symposium and following implementation. In the context of the new residency training program, we tracked the number of abstracts coauthored by categorical pediatrics residents, as well as those abstracts also presented at a regional or national meeting and those that were subsequently converted to peer-reviewed publications.

## Results

### Initial Needs Assessment

Prior to our first symposium in 2015, 30 faculty members completed the online needs assessment (Appendix A), a response rate of approximately 20%. Relevant to research symposium planning, we focused on responses to question five, “Please rank your level of confidence in the following skills,” rated on a 5-point Likert scale (1 = *not at all confident,* 5 = *extremely confident*). Faculty members gave an average response of 4.4 to both “My ability to teach medical students” and “My ability to teach residents.” The average response to “My ability to develop a scholarly project” was demonstrably lower at 3.2.

### Abstract Submissions and Studies

In our first year (2015), 29 abstracts were submitted by residents, faculty, and hospital associates. Abstracts increased in the second year to 46 and have remained fairly stable in years 3 and 4 at 56 and 51, respectively. There has also been an increase in research studies approved by our IRB since the inception of the symposium, from 65 in June 2015—before the first symposium—to 92 in summer 2016, to 123 in early 2019.

### Resident Scholarly Activity

Our first class of residents in the new categorical pediatrics program matriculated in 2015. During the first 4 years of the symposium, 61 abstracts were coauthored by residents from the first three classes in the program (*n* = 32). The same resident authors delivered 33 of the abstracts (54%) as oral or poster presentations at regional and national meetings. Fifteen of these abstracts (25%) were expanded to full manuscripts and subsequently published in the peer-reviewed literature.

### Attendance

In 2015, the initial symposium had 80 attendees, including residents, faculty members, and hospital associates. In 2016, that number remained stable at 80. In 2017, attendance increased to 100 participants, then to 150 in 2018.

### Evaluations: Quantitative and Qualitative Responses

Using individual session evaluations distributed on paper (Appendix J), 205 session evaluations were received in 2017 and 149 in 2018. The Table summarizes Likert-scale responses for the large-group sessions over these 2 years of the symposium. We prioritized the responses to the questions “I will apply what I learned during the session” and “This session should be held again next year” in establishing the perceived value of different sessions. Responses to these questions ranged from 4.6 to 5 (4 = *agree,* 5 = *strongly agree*). Workshops were evaluated individually, and data were distributed to the facilitators to assist them with their teaching development. Responses to the question “I will apply what I learned during this session,” aggregated across workshops, ranged from 4.7 to 5.

All evaluations included space for comments about the overall strengths and areas for improvement of the symposium. The most common narrative response themes are summarized below, with the number of evaluations including each theme in parentheses.

• Strengths of the symposium:
  ○ Opportunity for interaction, collegiality, networking (30 evaluations).
  ○ “2 Minutes, 2 Slides, 2 Questions” session (20 evaluations).
  ○ Learned new scholarly presentation or dissemination skills (18 evaluations).
  ○ Variety of content (17 evaluations).
  ○ Opportunity to practice new skills (14 evaluations).
  ○ Event was well organized (nine evaluations).
  ○ Received feedback on my work (seven evaluations).
• Areas for improvement:
  ○ Longer sessions, allow more time (eight evaluations).
  ○ Groups too close together/noise distraction (three evaluations).
  ○ Increase nonphysician representation/content (two evaluations).

## Discussion

We aimed to improve faculty and resident scholarship in both quantity and quality through the creation of an annual research symposium at our newly academically affiliated, community-based hospital. Accomplishing this aim would allow us to better care for patients by sharing clinical and professional lessons learned, to meet accreditation standards for the training programs, and to help faculty with career development. These goals were best addressed by merging our resources in research, education, and faculty development. We were able to develop this program with minimal funds by using freely available online resources and partnering with multiple groups in our facility.

The overall response has been excellent. Many residents used the experience of presenting at our local symposium to improve abstracts and posters that they subsequently submitted to regional and national meetings later in the academic year and to refine their presentations for eventual manuscript preparation. Faculty development was further enhanced by inviting faculty members to lead workshops, from which they gathered evaluations to use in support of their teaching portfolios and career development. Since 2015, 24 of our faculty and staff members have served as workshop leaders.

During this process, we have learned several valuable lessons about organizing a local academic event. First, adequate needs assessment and evaluation data are crucial for planning and improving future events to meet the needs of participants.

In 2015, the needs assessment completed by our teaching faculty (Appendix A, question 5) indicated high comfort levels with teaching learners and markedly lower comfort levels with scholarly project design and dissemination skills. We also had feedback from the ACGME—regarding our newly accredited residency training program—that our faculty scholarly productivity should be strengthened. We concluded that an event focused on sharing and developing scholarship within the hospital would be an effective way to address the gaps identified in the needs assessment. Since we started the symposium, the number of abstracts and research projects at our institution has markedly increased over 4 years.

For our symposium evaluations, we asked participants to complete electronic evaluations in 2015–2016 and had very poor response rates. Beginning in 2017, we found that a paper-based system worked best for our audience. Finding the most effective evaluation strategy is an ongoing question that future symposium leaders will need to address based on the local culture, environment, and resources.

We also noticed that fewer session evaluations were returned in 2018 than in 2017 despite an increased number of participants. This may have been due to some of the sessions offering evaluation forms in stacks at the door for participants to take on their way into the room. In 2017, we handed the evaluation forms to participants personally. This underscored to us the value of personal solicitation of evaluations in order to maximize response rates, as has been found in other educational programs (A. Gill, PhD, email, April 6, 2020).

Hosting the poster session initially posed a challenge as our institution did not own portable display boards or corkboards for traditional poster hanging using pushpins. These portable boards—or several dozen tripods—were prohibitively expensive to purchase, and we wanted to work with the available physical resources. We found that the use of poster putty and removable adhesive strips on wall surfaces was effective and inexpensive.

Comparing 2018 to 2017, the evaluations of the poster session were less favorable (Table). The narrative comments suggested that with increased numbers of high-quality posters, participants felt rushed to see them all and time became a limitation. In future years, we wish to maintain the high quality of the poster rounds while ensuring that participants can view an optimal number of posters. We are considering adjusting the time and reducing costs further by offering parts of the poster session as e-poster presentations, using screens to display posters on a rotation during the poster session. This would obviate the cost of printing traditional physical posters. The limitation of this approach is the technology required, and we are still examining the resources available for this purpose.

**Table.**
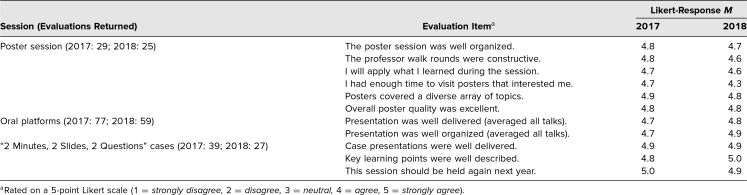
Symposia Evaluation Responses From 2017–2018

Evaluation data have also been especially helpful in topic selection and facilitation of workshops. From 2017 to 2018, we retained two workshop topics—grant writing and quality improvement—based on evaluations. The grant writing workshop presenters used corrective feedback from 2017 to substantially restructure the workshop, and this was reflected in greater participant satisfaction on their evaluations in 2018. The quality improvement workshop facilitators were able to maintain high quality as measured by evaluations during both years.

Our direct evaluation data have been limited thus far to participant satisfaction and immediate knowledge and attitudes following participation. However, we have also sought to identify changes in behavior our participants make as a result of the symposium. Largely indirect measures of improved research motivation or skills have included participation in the symposium, number of IRB submissions during the initial years of the symposium, and wider dissemination of resident work arising from the symposium; these data were suggestive of an impactful experience (see Results). Future efforts at outcomes assessment will include offering delayed evaluation to participants several months after the symposium to examine whether and how research behaviors have changed as a result of participation.

We have also felt challenged to increase the diversity of participants at our symposium. While we set out initially to benefit faculty and trainee scholarship, we have over the past 2 years set a goal of increasing participation by other hospital associates, including nursing, chaplains, child life specialists, dietitians, and pharmacists. Each year, we have some participants from these groups, but recruiting and encouraging them remains a challenge. This issue was raised as a comment in only two of the participant surveys (see Results), but the paucity of this feedback only underscores the limited participation of nonphysician, nontrainee groups. In coming years of the symposium, we are including a nursing leader in our planning committee in order to broaden the audience and demonstrate intentionality for increased inclusiveness.

Another limitation that symposium leaders may face relates to available space. Our hospital's education space encompasses half of one floor of our clinic building, and we are gradually outgrowing it. We have found that we can expand our poster viewing area by hanging posters not only in the assigned meeting rooms but also in the foyer and hallways surrounding this space. The growth process has required ongoing creativity.

Finally, and possibly most importantly, funding is a perennial concern for symposium events. As detailed in the Methods section, above, we have been able to defray costs by building partnerships between different groups in the hospital. Organizers at other institutions could start with our current budget (Appendix I) and potentially eliminate items if needed. For example, if monetary poster awards are not feasible, printed certificates may suffice. If funds are not available for travel and lodging for a national speaker, a local or regional leader may be willing to serve as a keynote or grand rounds speaker without incurring costs. Provision of meals by the symposium is not essential and could be eliminated to save money, although we suspect that providing lunch during the poster session may have improved attendance.

Using our materials, we expect that leaders in other community-based clinical settings will be empowered to develop their own local symposia grounded in their programs' specific needs and resources. This process can help kindle academic enthusiasm among faculty and trainees and provide needed scholarly accomplishments for maintenance of program accreditation.

## Appendices

Needs Assessment.docxSample Symposium Agenda.docxSymposium Planning Checklist.docxAbstract Submission Form.docxAbstract Quality Scoring Rubric.docxCorrespondence With Abstract Authors.docxPoster Session Moderator Instructions.docxPoster Session Moderator Scoring Sheet.docxSample Budget.docxSample Symposium Session Evaluation Forms.docx
All appendices are peer reviewed as integral parts of the Original Publication.
