# Supplementary material for: Building a Local Research Symposium: The Crossroads of Scholarship, Education, and Faculty Development
Source: MedEdPORTAL. 2020 Dec 24;16:11048. doi: 10.15766/mep_2374-8265.11048 (PMC7780738; doi:10.15766/mep_2374-8265.11048)
Supplement: Supplementary file 1 — Needs Assessment.docxSample Symposium Agenda.docxSymposium Planning Checklist.docxAbstract Submission Form.docxAbstract Quality Scoring Rubric.docxCorrespondence With Abstract Authors.docxPoster Session Moderator Instructions.docxPoster Session Moderator Scoring Sheet.docxSample Budget.docxSample Symposium Session Evaluation Forms.docx [file mep_2374-8265.11048-s001.zip › A. Needs Assessment.docx]

**Appendix A**

**Needs Assessment**

***Note:*** *The following needs assessment may be built into any online survey tool of choice. Adjust the questions to meet the institution’s current practice and learner audience as needed.*

**Instructions:** Please take 5-10 minutes to provide your perspectives on our Faculty Development program. Your responses are anonymous, and will help to craft the upcoming year’s topics and schedule.

1. How long has it been since you completed your last year of training (residency or fellowship)?
   - 0-4 years
   - 5-9 years
   - 10+ years
2. Which of the following best describes your clinical role? Select as many as apply for you.
   - Primary care
   - Hospital Medicine
   - Critical Care Medicine (including neonatology)
   - Emergency Medicine
   - Subspecialty
3. During this past academic year, what are the key barriers you have experienced to participating in Faculty Development events? Select all that apply.
   - I was not aware of events
   - I forgot or became too busy to attend events although they were in my calendar
   - Events were held at a bad time for me
   - I was not interested in one or more of the topics offered
   - I considered myself sufficiently knowledgeable or experienced in one or more of the topics offered
   - Other: __________________________

**Identifying Opportunities for Improvement:** These questions seek information regarding the ongoing goals and structure of our Faculty Development curriculum.

1. We currently hold Faculty Development sessions monthly on Fridays during the noon hour. Please indicate your level of agreement with each of these statements:

|  | **Strongly Disagree** | **Disagree** | **Neither Agree nor Disagree** | **Agree** | **Strongly Agree** |
| --- | --- | --- | --- | --- | --- |
| I am satisfied with my ability to attend the current Friday noon sessions. | ⃝ | ⃝ | ⃝ | ⃝ | ⃝ |
| I would find it easier to attend Faculty Development if it was offered in the early mornings. | ⃝ | ⃝ | ⃝ | ⃝ | ⃝ |
| I would find it easier to attend Faculty Development if it was offered late in the afternoons. | ⃝ | ⃝ | ⃝ | ⃝ | ⃝ |
| I would be able to make time to attend longer sessions that are more workshop-based, provided I have advance notice. | ⃝ | ⃝ | ⃝ | ⃝ | ⃝ |
| Offering Faculty Development on a monthly basis is an appropriate frequency. | ⃝ | ⃝ | ⃝ | ⃝ | ⃝ |
| Having lunch provided is an important motivator to my attending Faculty Development. | ⃝ | ⃝ | ⃝ | ⃝ | ⃝ |

1. Please rank your level of confidence in the following skills:

|  | **Not at all confident** | **Somewhat lacking confidence** | **Neither confident nor lacking confidence** | **Somewhat confident** | **Very confident** |
| --- | --- | --- | --- | --- | --- |
| My ability to teach residents in clinical settings | ⃝ | ⃝ | ⃝ | ⃝ | ⃝ |
| My ability to teach medical students in clinical settings | ⃝ | ⃝ | ⃝ | ⃝ | ⃝ |
| My ability to present a didactic conference for residents | ⃝ | ⃝ | ⃝ | ⃝ | ⃝ |
| My ability to develop a scholarly project | ⃝ | ⃝ | ⃝ | ⃝ | ⃝ |
| My ability to disseminate a scholarly project (e.g., abstract, poster, manuscript) | ⃝ | ⃝ | ⃝ | ⃝ | ⃝ |

1. We currently aim for a 50/50 balance of education and scholarship topics in our Faculty Development series. Please indicate what you feel is the best balance between these topic areas.

- We should focus entirely on education topics.
- We should emphasize education topics, but also include some sessions on scholarship.
- We should continue to pursue an equal balance between education and scholarship topics.
- We should emphasize scholarship topics, but also include some sessions on education.
- We should focus entirely on scholarship topics.
- I am not sure or have no opinion.

1. Please list one or more topics or questions you would like to see addressed in the coming year during Faculty Development (you may also request repeats of topics we have covered in the past).

______________________________________________________________________________

______________________________________________________________________________

______________________________________________________________________________

1. Please offer any other comments about Faculty Development at our institution that you feel could help us to plan the coming year’s curriculum.

______________________________________________________________________________

______________________________________________________________________________

______________________________________________________________________________
