# Supplementary material for: Building a Local Research Symposium: The Crossroads of Scholarship, Education, and Faculty Development
Source: MedEdPORTAL. 2020 Dec 24;16:11048. doi: 10.15766/mep_2374-8265.11048 (PMC7780738; doi:10.15766/mep_2374-8265.11048)
Supplement: Supplementary file 1 — Needs Assessment.docxSample Symposium Agenda.docxSymposium Planning Checklist.docxAbstract Submission Form.docxAbstract Quality Scoring Rubric.docxCorrespondence With Abstract Authors.docxPoster Session Moderator Instructions.docxPoster Session Moderator Scoring Sheet.docxSample Budget.docxSample Symposium Session Evaluation Forms.docx [file mep_2374-8265.11048-s001.zip › B. Sample Symposium Agenda.docx]

**Appendix B**

**Sample symposium agenda**

**Symposium date:** _______________________

**Instructions:** Modify the agenda below based on local content needs assessment, available resources, and number of presenters.

| **Time** | **Session Title** | **Session Description** |
| --- | --- | --- |
| 7:30 – 8:30 a.m. | Grand Rounds | Address a needed theme in scholarship, if an invited or keynote speaker is available |
| 8:40 – 10:10 a.m. | Concurrent Workshops I | Select workshops based on needs assessment and faculty expertise; suggestions include:   - Case report authorship - Effective abstract writing - Effective poster design - How to write Institutional Review Board proposals - How to write pilot grant proposals - Human subjects protection and informed consent - Manuscript authorship - Project management - Quality improvement project design - Research survey design |
| 10:20 a.m. – 12:00 p.m. | Oral Session I | Invited abstract platform presentations; suggested schedule includes 15 minutes per presentation, 5 minutes for questions (5 presentations = 80 minutes) |
| 12:00 – 1:30 p.m. | Poster Session | Author-attended poster presentations, professor walk rounds at poster session (see instructions); serve lunch if available, provide lunch break if needed |
| 1:30 – 3:10 p.m. | Oral Session II | Additional invited abstract platform presentations as above; 2 minutes/2 slides/2 questions clinical case symposium (see instructions); suggested schedule includes 2 additional platform talks (40 minutes) plus 15 short case presentations (60 minutes) |
| 3:20 – 4:50 p.m. | Concurrent Workshops II | Offer additional workshops as desired; see suggestions above |
