# Supplementary material for: Building a Local Research Symposium: The Crossroads of Scholarship, Education, and Faculty Development
Source: MedEdPORTAL. 2020 Dec 24;16:11048. doi: 10.15766/mep_2374-8265.11048 (PMC7780738; doi:10.15766/mep_2374-8265.11048)
Supplement: Supplementary file 1 — Needs Assessment.docxSample Symposium Agenda.docxSymposium Planning Checklist.docxAbstract Submission Form.docxAbstract Quality Scoring Rubric.docxCorrespondence With Abstract Authors.docxPoster Session Moderator Instructions.docxPoster Session Moderator Scoring Sheet.docxSample Budget.docxSample Symposium Session Evaluation Forms.docx [file mep_2374-8265.11048-s001.zip › C. Symposium Planning Checklist.docx]

**Appendix C**

**Symposium Planning Checklist**

**Symposium date:** _______________________

**Tasks to complete ≥ 3 months prior to symposium date:**

| ✔ | **Task Description** | **Timeframe** | **Due date** |
| --- | --- | --- | --- |
| ⬜ | Needs assessment / review of prior evaluations | 9-12 months ahead | _________________ |
| ⬜ | Select date – ensure no conflict with regional or national meetings that stakeholders might attend | 9-12 months ahead | _________________ |
| ⬜ | Reserve necessary space | 9-12 months ahead | _________________ |
| ⬜ | Identify and invite Keynote / Grand Rounds speaker | 6-12 months ahead | _________________ |
| ⬜ | Send “save the date” announcements to all potential participants | 6 months ahead | _________________ |
| ⬜ | Budgeting / secure funding | 6 months ahead | _________________ |
| ⬜ | Solicit workshop proposals from faculty / invite specific workshop facilitators | 3-6 months ahead | _________________ |
| ⬜ | Catering reservations if providing meals | 3 months ahead | _________________ |
| ⬜ | Ensure guest speaker (if traveling) has travel and hotel arrangements | 3 months ahead | _________________ |

**Tasks to complete 1 month – 3 months prior to symposium date:**

| ✔ | **Task Description** | **Timeframe** | **Due date** |
| --- | --- | --- | --- |
| ⬜ | Prepare abstract submission – online form or by email | 2-3 months ahead | _________________ |
| ⬜ | Send out call for abstract submissions | 2-3 months ahead | _________________ |
| ⬜ | Send preliminary symposium agenda to all potential participants | 1-2 months ahead | _________________ |
| ⬜ | Invite faculty colleagues to serve as poster moderators, instructions and abstracts to come after review | 2 months ahead | _________________ |
| ⬜ | Abstract submission deadline | ~ 5 weeks ahead | _________________ |
| ⬜ | Send submitted abstracts and scoring rubric to reviewers for individual scoring | ~ 5 weeks ahead | _________________ |
| ⬜ | Reviewers meet to select abstracts for oral and poster invitation | ~ 1 month ahead | _________________ |
| ⬜ | Order/purchase poster hanging supplies | ~1 month ahead | _________________ |

**Tasks to complete 1-4 weeks prior to symposium date:**

| ✔ | **Task Description** | **Timeframe** | **Due date** |
| --- | --- | --- | --- |
| ⬜ | Notification emails to invited poster and oral presenters | 4 weeks ahead | _________________ |
| ⬜ | Preparation of documents:  ⬜ Design program or abstract book  ⬜ Evaluation forms – paper or electronic  ⬜ Signage (if needed)  ⬜ Space numbers for poster hanging  ⬜ Sign-in sheets for each session |  | _________________  _________________  _________________  _________________  _________________ |
| ⬜ | Dinner reservations if hosting a visiting speaker | 2-4 weeks ahead | _________________ |
| ⬜ | Number poster abstracts | 2 weeks ahead | _________________ |
| ⬜ | Send out poster session moderator assignments and instructions | 2 weeks ahead | _________________ |

**Tasks to complete 1 week ahead to the day of the symposium:**

| ✔ | **Task Description** | **Timeframe** | **Due date** |
| --- | --- | --- | --- |
| ⬜ | Collect oral presentations from speakers | 1-7 days ahead | _________________ |
| ⬜ | Finalize program / abstract book | 1-7 days ahead | _________________ |
| ⬜ | Confirm poster session moderators | 1-7 days ahead | _________________ |
| ⬜ | Final communication with presenters about the time of presentations / where and when to hang posters | 1-7 days ahead | _________________ |
| ⬜ | Hang posters | Symposium day | _________________ |
| ⬜ | Huddle with poster session moderators to review instructions and assignments | Symposium day | _________________ |
| ⬜ | Recruit volunteers from attendees to keep track of time during poster session walk rounds | Symposium day | _________________ |
| ⬜ | Collect scoring sheets from poster session moderators, decide on awards (if giving) | Symposium day | _________________ |
| ⬜ | Collect session evaluations | Symposium day | _________________ |

**Tasks to complete after the symposium:**

| ✔ | **Task Description** | **Timeframe** | **Due date** |
| --- | --- | --- | --- |
| ⬜ | Collate session evaluations, share with presenters of various sessions | 1-2 weeks after | _________________ |
| ⬜ | Thank partners / funders | 1-2 weeks after | _________________ |
| ⬜ | Leadership team meet to debrief, take notes on future changes based on discussion and evaluations | 1-4 weeks after | _________________ |
