# Supplementary material for: Building a Local Research Symposium: The Crossroads of Scholarship, Education, and Faculty Development
Source: MedEdPORTAL. 2020 Dec 24;16:11048. doi: 10.15766/mep_2374-8265.11048 (PMC7780738; doi:10.15766/mep_2374-8265.11048)
Supplement: Supplementary file 1 — Needs Assessment.docxSample Symposium Agenda.docxSymposium Planning Checklist.docxAbstract Submission Form.docxAbstract Quality Scoring Rubric.docxCorrespondence With Abstract Authors.docxPoster Session Moderator Instructions.docxPoster Session Moderator Scoring Sheet.docxSample Budget.docxSample Symposium Session Evaluation Forms.docx [file mep_2374-8265.11048-s001.zip › D. Abstract Submission Form.docx]

**Appendix D**

**Abstract Submission Form**

*The following questions were formatted using a free Google Forms account. The same content could be built into any desired online survey/form collecting platform.*

***Note:*** *The Google Forms platform allows limitation of the abstract submission by number of characters (not number of words). This form was built to limit abstract text to 2,500 characters; at an average length of English words of just over 5 characters, this limitation comes to under 500 words. This is customizable based on user’s need.*

**Abstract Submission: Research Symposium**

**Deadline: _______________**

**Instructions:** Fill out this complete form separately for each abstract you are submitting. There will be a link to submit additional abstracts when you complete the form.

**Please note:** All communication regarding your abstract will be to the corresponding author. The corresponding author is responsible for sharing information with all co-authors. Please make sure the contact information you enter below is accurate and your preferred email address.

(* = Required fields)

1. Email address *

____________________________

1. Corresponding Author’s name (include any applicable degrees or titles) *

____________________________

1. Author names * (List ALL author names and affiliations, including the above Corresponding Author, in the desired order of authorship. Please use the following format for consistency when we print the abstracts: Lastname Initials, affiliation; next author. Example: Jones YN, Children’s Hospital; Livingstone S, Healthcare Associates; etc.)

______________________________________________________________________________

1. Abstract title *

______________________________________________________________________________

1. Abstract text * (Type or paste your abstract below. Special characters and formatting may not be preserved, so please proofread carefully. Please limit your abstract to 2,500 characters [approximately 350-400 words] or fewer. It may be structured or unstructured. For structured abstracts, we recommend the IMRaD structure [introduction/background, methods, results, and discussion/conclusion]. Please do not include figures, tables, footnotes, or references.)

______________________________________________________________________________

1. Please consider this abstract for: *
   1. Poster presentation only
   2. Oral presentation only
   3. Both poster and oral presentation
2. This abstract fits into the following category(ies) of scholarship. Select all that apply: *
   1. Basic science research
   2. Translational research
   3. Clinical research
   4. Quality improvement/patient safety
   5. Education
   6. Advocacy
   7. Community health, global health, or epidemiology
   8. Clinical conundrum or case report
   9. Other: __________________________
