# Supplementary material for: Building a Local Research Symposium: The Crossroads of Scholarship, Education, and Faculty Development
Source: MedEdPORTAL. 2020 Dec 24;16:11048. doi: 10.15766/mep_2374-8265.11048 (PMC7780738; doi:10.15766/mep_2374-8265.11048)
Supplement: Supplementary file 1 — Needs Assessment.docxSample Symposium Agenda.docxSymposium Planning Checklist.docxAbstract Submission Form.docxAbstract Quality Scoring Rubric.docxCorrespondence With Abstract Authors.docxPoster Session Moderator Instructions.docxPoster Session Moderator Scoring Sheet.docxSample Budget.docxSample Symposium Session Evaluation Forms.docx [file mep_2374-8265.11048-s001.zip › E. Abstract Quality Scoring Rubric.docx]

**Appendix E**

**Abstract Quality Scoring Rubric**

**Lead Author: __________________________**

**Title:** _______________________________________________________________________________

**Evaluator:** __________________________

| **Category description** | **Score** |
| --- | --- |
| Introduction – Is background information presented on topic? What is known and unknown, how will this abstract fill the gap? | - 0 = missing - 1 = poorly explained - 2 = partially explained - 3 = partially explained but well written - 4 = well explained |
| Hypothesis – Is it clearly stated? | - 0 = missing - 1 = present, but vague - 2 = well stated |
| Methods – Are study parameters clearly identified and appropriately chosen to address hypothesis, e.g., retrospective/prospective, randomized or not, observation/interventional, population studied, appropriate design, data analysis methods, etc.? | - 0 = missing - 1 = poorly explained - 2 = partially explained and method not well selected - 3 = partially explained or method not well selected - 4 = well explained and appropriate method |
| Results – Do results match selected methods, is data interpretation/statistical approach appropriate? Are results clearly summarized? | - 0 = missing - 1 = partially explained based on criteria to the left - 2 = partially explained based on criteria to the left - 3 = partially explained based on criteria to the left - 4 = well explained |
| Conclusion – Is it appropriately stated? Is there overgeneralization? Does it match hypothesis/aims? Is there clinical relevance? | - 0 = missing - 1 = partially addressed criteria on the left - 2 = partially addressed criteria on the left - 3 = partially addressed criteria on the left - 4 = clear, appropriate, addresses hypothesis, relevant |
| Total | ___________ / 18 |
