# Supplementary material for: Building a Local Research Symposium: The Crossroads of Scholarship, Education, and Faculty Development
Source: MedEdPORTAL. 2020 Dec 24;16:11048. doi: 10.15766/mep_2374-8265.11048 (PMC7780738; doi:10.15766/mep_2374-8265.11048)

**Appendix F**

**Correspondence with abstract authors**

Emails to abstract authors can be prepared manually or automatically using various software options. Manual emails of acceptances of oral and poster presentations may be more feasible for smaller numbers of submissions, and may become more time consuming with increased numbers. Suggestions for automation of correspondence are included at the end of this Appendix.

Sample email templates (change the highlighted date/time information in the first paragraph depending on timing of symposium):

1. Invited oral presentation

Dear **«Corresponding_Authors_Name»**,

On behalf of the Research Symposium Planning Committee, I am pleased to invite you to present your project, entitled **“«Abstract_title»,”** as an oral platform presentation at our Research Symposium on [date]. We had 50 excellent submissions this year in our call for abstracts, and selected 7 for platform presentations. The platform talks will be held during morning and afternoon sessions, your time slot will be assigned in [timeframe].

For your platform presentation, you will have up to 15 minutes to present, and up to 5 minutes after that for questions (20 minutes total per presenter). You are also invited to present your work as a poster during the poster session that day.

Please reply to this email as soon as possible with the following information:

1. I accept the invitation to present: Y/N
2. I will also present this abstract as a poster (we want to have an accurate poster count to allocate space): Y/N

Also, please share this information with any co-authors of this abstract. I will continue to correspond with you as the Symposium approaches with further instructions and information.

Thank you for your outstanding submission, and we are looking forward to hearing more about your work!

1. Invited poster presentation

Dear **«Corresponding_Authors_Name»**,

On behalf of the Research Symposium Planning Committee, I am pleased to invite you to present your project, entitled **“«Abstract_title»,”** during the poster session at our Research Symposium on [date]. The author-attended poster session will be held from [time].

Please reply to this email as soon as possible to confirm your poster presentation (we want to have an accurate poster count to allocate space).

Also, please share this information with any co-authors of this abstract. I will continue to correspond with you as the Symposium approaches with further instructions and information.

Thank you for your outstanding submission, and we are looking forward to hearing more about your work!

**Regarding automation of email correspondence**

Google Docs is a freely available word processing tool online, which can be augmented with a “mail merge” feature. Users of this service must navigate to the “Add Ons” menu and search the available Add On tools for “mail merge.” There are several tools available by this search, which come with instructions for users to automate their email messages using a spreadsheet of on-line responses.

A tutorial video on the use of Google Docs and Google Sheets to perform this function is available at: <https://www.youtube.com/watch?v=iHK4ASwHpiI>

For users of Microsoft Word, the merge tools are available within the main product. Sample instructions are included below. Note that while these tools have always been available in prior and current versions of MS Word, it is possible that future editions of the software will have different menu appearances or change these features.

**Microsoft Word mail merge steps and sample emails to invited oral and poster abstract authors**

1. Download spreadsheet of abstract submissions from web-based form (e.g., Google Forms, SurveyMonkey, etc.) using the host site’s instructions. The file format should be *.csv, *.xls, *xlsx
2. Open a blank MS Word document. Link the document to the spreadsheet of submissions using the “Mailings” tab, “Select Recipients,” and “Use an Existing List” options as shown below.


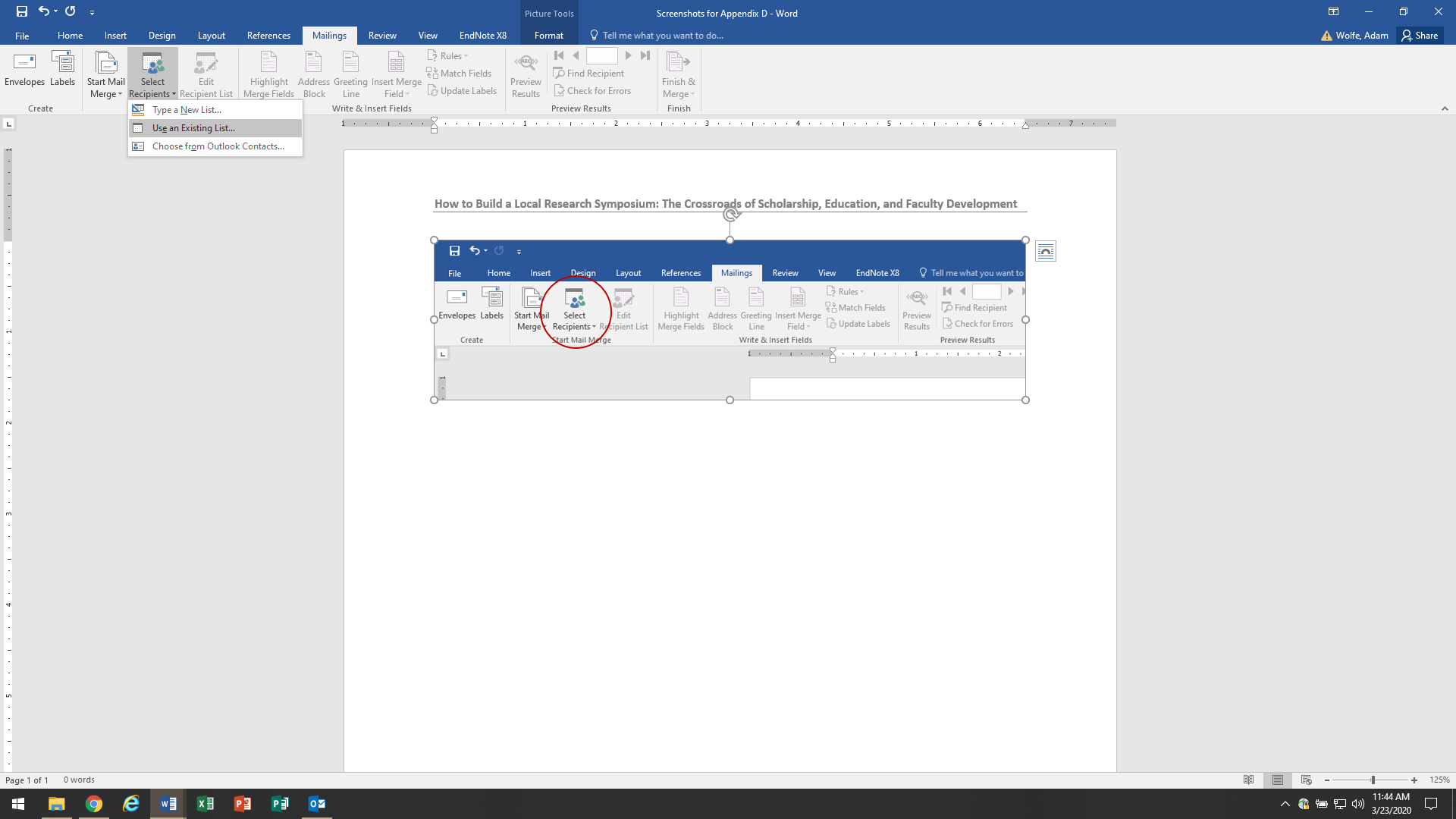

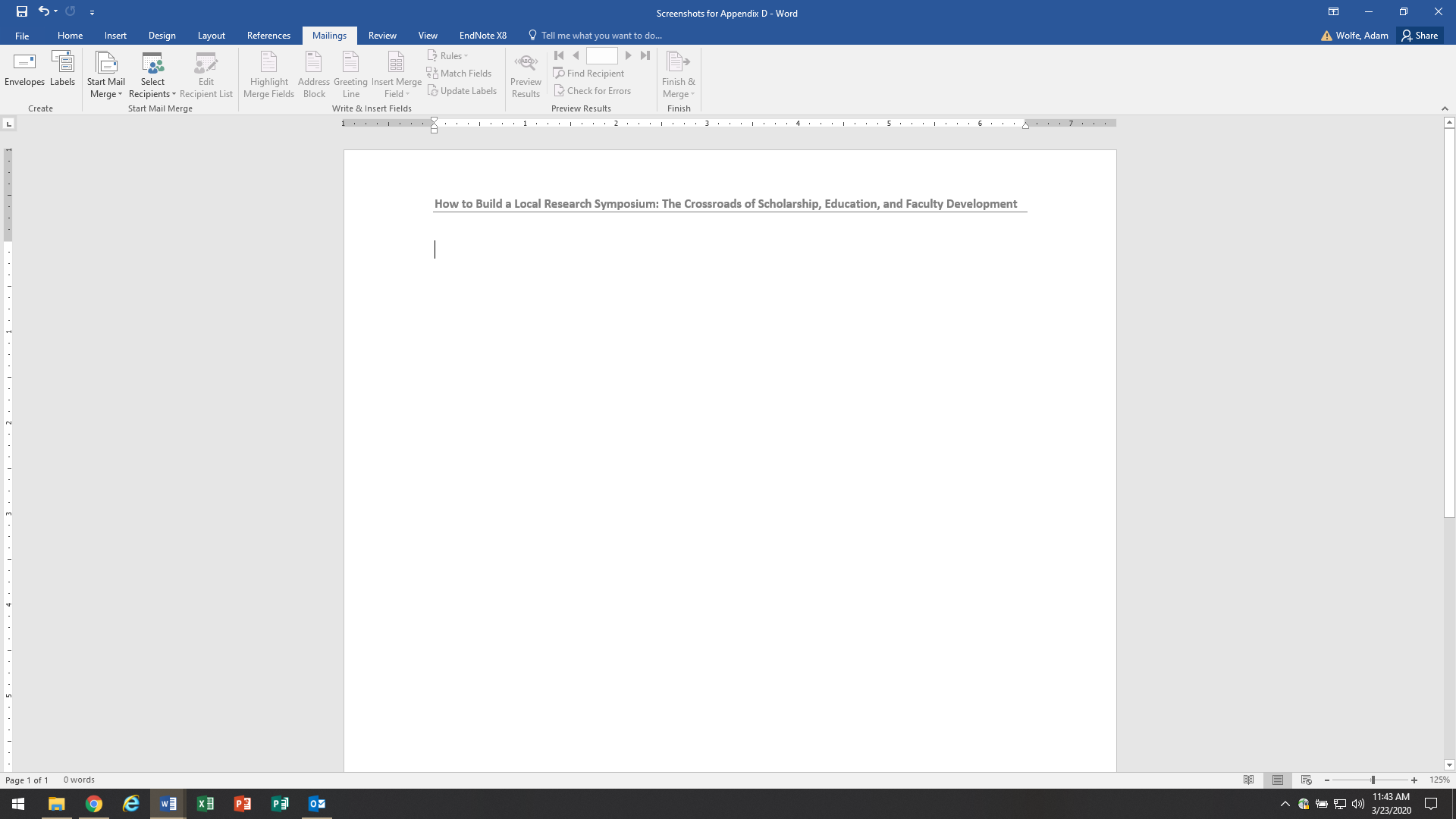


1. Use the file browser to select the spreadsheet of abstract submissions.
2. Begin constructing emails to authors using the templates above. For the author name, abstract title, and any other information specific to the submission, use the “Insert Merge Field” instruction. It should bring up a listing of all columns from the submission spreadsheet; select the desired field(s) from the spreadsheet as shown below. The field name will appear in the email; these are bolded in the templates that follow.


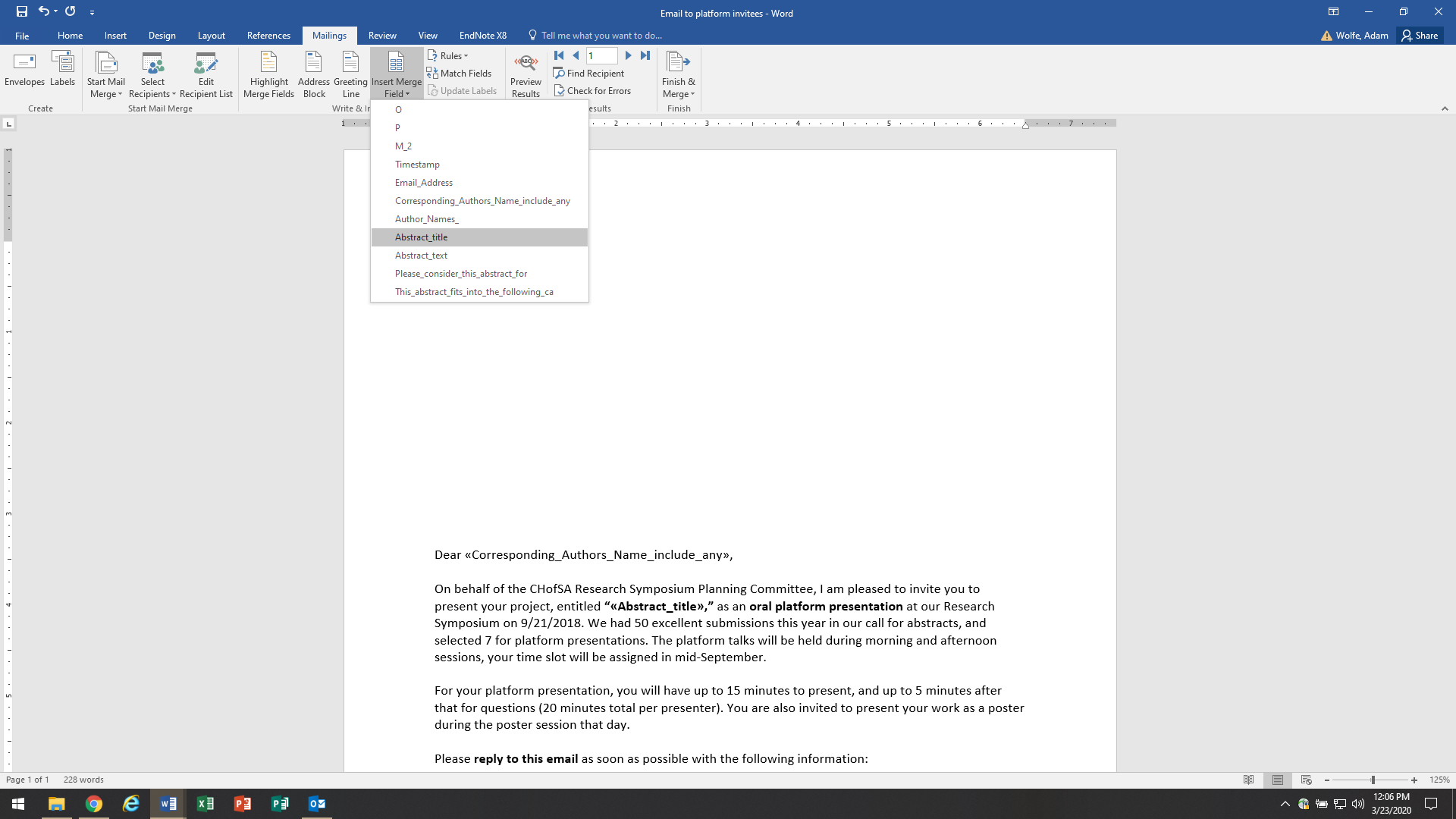


1. Select the desired recipients. If all abstract authors should be receiving an email, no extra steps are required. If the email is only going to a subset of authors (e.g., invited oral presentations), then follow the instructions below to select recipients.
   1. Select “Edit Recipient List” (Circle “1” in image below)
   2. Uncheck box at the top of 2^nd^ column to deselect all (Circle “2” in image below)
   3. Individually check all desired recipients of this email in the 2^nd^ column (Circle “3” in image below)


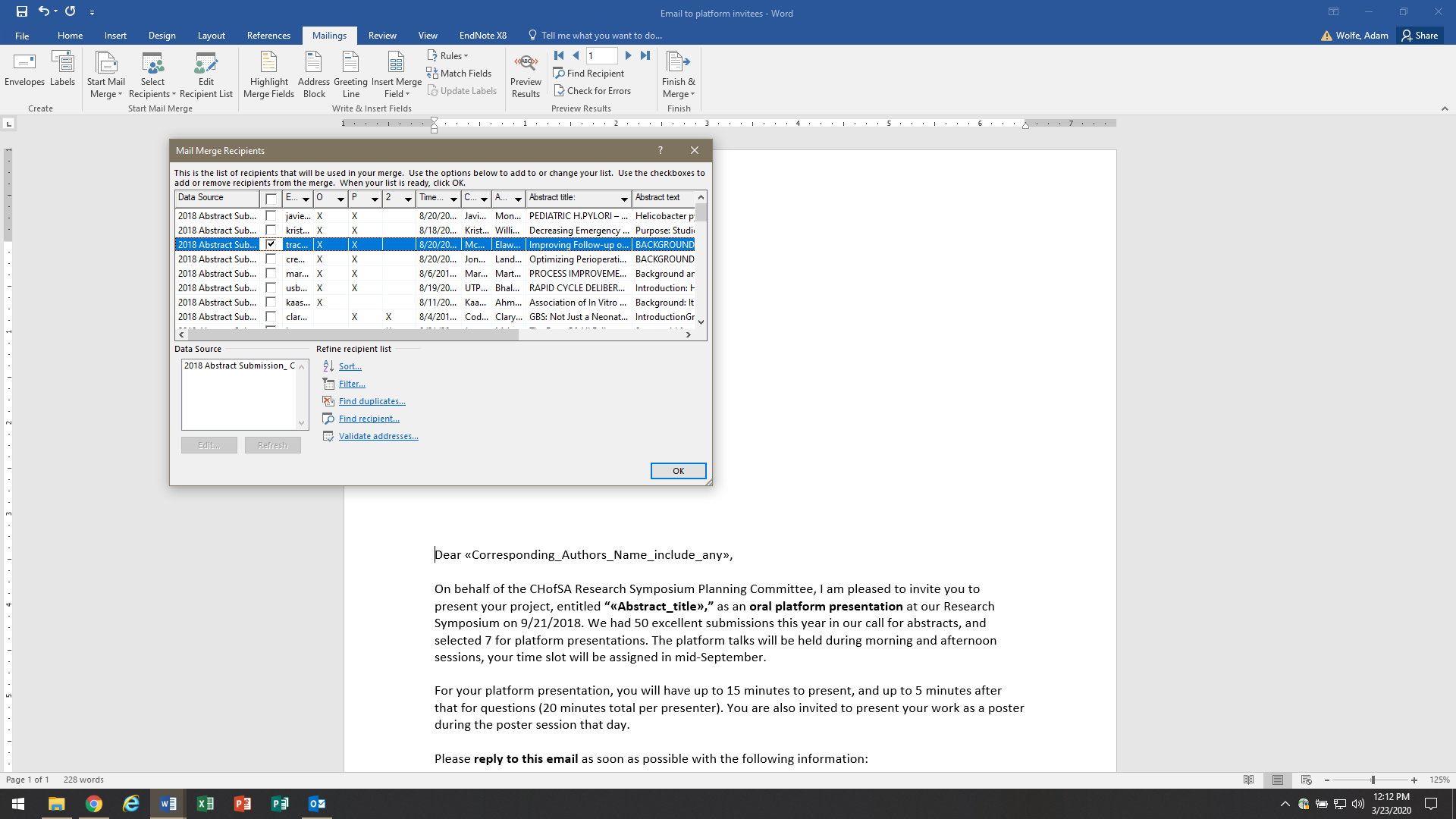


1. Optional: Create proofs of emails. To view how the final, merged email messages will look before sending, navigate to “Finish & Merge,” and select “Edit Individual Documents” from the drop-down list. This will create a new document with each individual email on a separate page, allowing a quality check of whether the merge fields inserted correctly into the messages. This new document will not send the emails.
   1. If no errors are identified, close the new document to return to the working template and proceed to step 9.
   2. If errors are identified, close the new document, return to the working template, and make any desired edits starting from step 4 above.


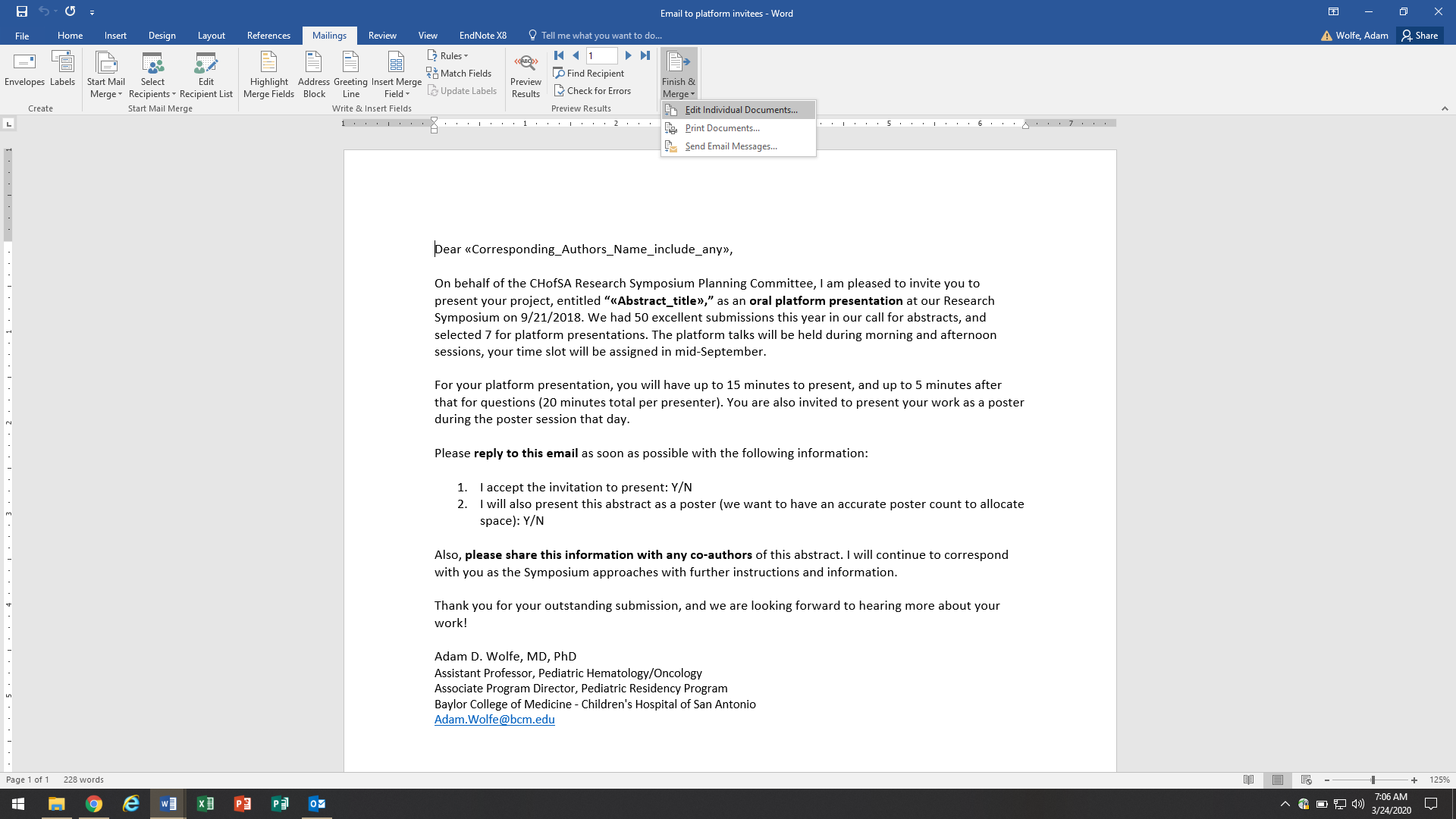


1. Send the emails. This step assumes that a user’s Microsoft Office is linked to an email client such as MS Outlook. Under “Finish & Merge” select “Send Email Messages” and the emails will individually go out from the user’s account. This can be confirmed by opening the email client and the messages should appear in the “Sent” folder.


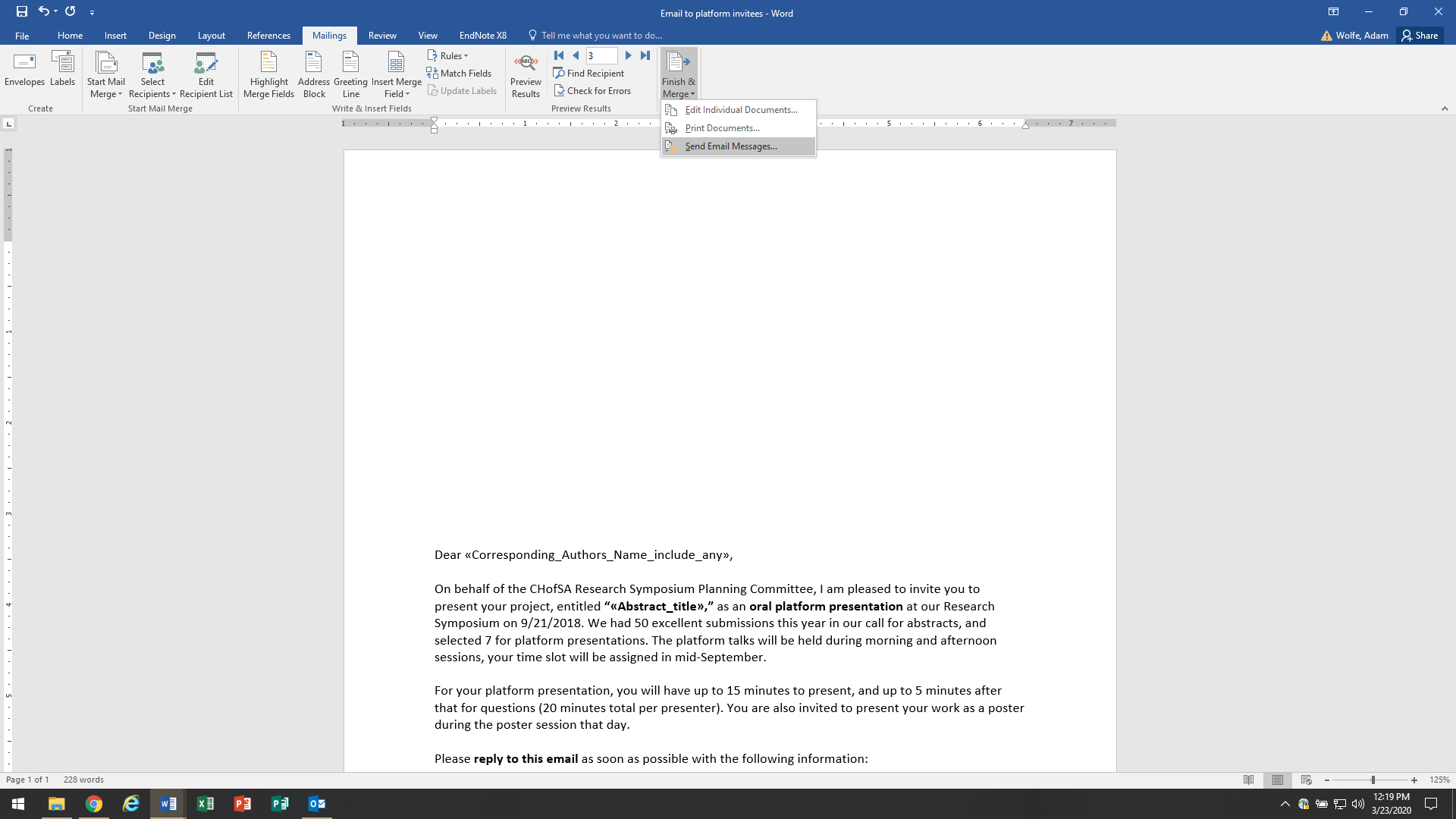

Supplement: Supplementary file 1 — Needs Assessment.docxSample Symposium Agenda.docxSymposium Planning Checklist.docxAbstract Submission Form.docxAbstract Quality Scoring Rubric.docxCorrespondence With Abstract Authors.docxPoster Session Moderator Instructions.docxPoster Session Moderator Scoring Sheet.docxSample Budget.docxSample Symposium Session Evaluation Forms.docx [file mep_2374-8265.11048-s001.zip › F. Correspondence with Abstract Authors.docx]
