# Supplementary material for: Building a Local Research Symposium: The Crossroads of Scholarship, Education, and Faculty Development
Source: MedEdPORTAL. 2020 Dec 24;16:11048. doi: 10.15766/mep_2374-8265.11048 (PMC7780738; doi:10.15766/mep_2374-8265.11048)
Supplement: Supplementary file 1 — Needs Assessment.docxSample Symposium Agenda.docxSymposium Planning Checklist.docxAbstract Submission Form.docxAbstract Quality Scoring Rubric.docxCorrespondence With Abstract Authors.docxPoster Session Moderator Instructions.docxPoster Session Moderator Scoring Sheet.docxSample Budget.docxSample Symposium Session Evaluation Forms.docx [file mep_2374-8265.11048-s001.zip › H. Poster Session Moderator Scoring Sheet.docx]

**Appendix H**

**Poster session moderator scoring sheet**

**Poster Presentations**

**Judge’s Score Sheet and Evaluation**

PRESENTER’S NAME_________________________POSTER No.__________________________________

***Subject Matter and Scientific Merit*** (1 = Poor, 3 = Satisfactory, 5 = Excellent)

| Title Descriptive | 1 | 2 | 3 | 4 | 5 |
| --- | --- | --- | --- | --- | --- |
| Introduction/Background (sets poster context) | 1 | 2 | 3 | 4 | 5 |
| Objectives clearly stated | 1 | 2 | 3 | 4 | 5 |
| Methods/Approach (adequate to address objectives; appropriate details) | 1 | 2 | 3 | 4 | 5 |
| Results well summarized (tied back to objectives) | 1 | 2 | 3 | 4 | 5 |
| Conclusions (take home messages) | 1 | 2 | 3 | 4 | 5 |
| Originality (novel methods, new approach) | 1 | 2 | 3 | 4 | 5 |
| Scientific/Management Merit (valuable results and cogent interpretation) | 1 | 2 | 3 | 4 | 5 |
| TOTAL SCORE FOR THIS CATEGORY (40 possible points) |  |  |  |  |  |

***Poster Style, Organization, and Visuals***

| Organization (logical presentation and progression of ideas) | 1 | 2 | 3 | 4 | 5 |
| --- | --- | --- | --- | --- | --- |
| Flow (logical layout and can follow easily from one section to next) | 1 | 2 | 3 | 4 | 5 |
| Visual appeal 9attractive and balanced) | 1 | 2 | 3 | 4 | 5 |
| Legibility (appropriate font, heading, captions, neat and easy to read text) | 1 | 2 | 3 | 4 | 5 |
| Figures and Tables (simple, illustrative, tied to results) | 1 | 2 | 3 | 4 | 5 |
| Illustrations (eye-catching artwork or photography tied to results) | 1 | 2 | 3 | 4 | 5 |
| Authors and contributors (location and funding sources easy to identify) | 1 | 2 | 3 | 4 | 5 |
| Writing clarity (easy to understand, short and direct statements) | 1 | 2 | 3 | 4 | 5 |
| Message (easy to quickly gather implications of work) | 1 | 2 | 3 | 4 | 5 |
| Volume of material (appropriate amount of information for a poster) | 1 | 2 | 3 | 4 | 5 |
| TOTAL SCORE FOR THIS CATEGORY (50 possible points) |  |  |  |  |  |

***Interaction with poster viewers and reviewers***

| Can summarize poster thoroughly and succinctly | 1 | 2 | 3 | 4 | 5 |
| --- | --- | --- | --- | --- | --- |
| Answers questions briefly but thoroughly | 1 | 2 | 3 | 4 | 5 |
| TOTAL SCORE FOR THIS CATEGORY (10 possible points) |  |  |  |  |  |

***Comments***

MODERATOR NAME: ___________________________________

SIGNATURE: ___________________________________

FINAL SCORE: ________________
