# Supplementary material for: Building a Local Research Symposium: The Crossroads of Scholarship, Education, and Faculty Development
Source: MedEdPORTAL. 2020 Dec 24;16:11048. doi: 10.15766/mep_2374-8265.11048 (PMC7780738; doi:10.15766/mep_2374-8265.11048)
Supplement: Supplementary file 1 — Needs Assessment.docxSample Symposium Agenda.docxSymposium Planning Checklist.docxAbstract Submission Form.docxAbstract Quality Scoring Rubric.docxCorrespondence With Abstract Authors.docxPoster Session Moderator Instructions.docxPoster Session Moderator Scoring Sheet.docxSample Budget.docxSample Symposium Session Evaluation Forms.docx [file mep_2374-8265.11048-s001.zip › I. Sample Budget.docx]

**Appendix I**

**Sample Budget**

| **Category** | **Line Item** | **Quantity** | **Budget Cost (Unit/Total)** | **Actual Cost (Unit/Total)** |
| --- | --- | --- | --- | --- |
| Travel | Grand Rounds Speaker Airfare | 1 | $500/500 | $621.64/621.64 |
| Lodging | Grand Rounds Speaker Hotel | 1 | $150/150 | $170.56/170.56 |
| Food | Lunch at Poster Session | 100 | $8/800 | $6.50/650 |
|  | Dinner for Grand Rounds Speaker | 1 | $200/200 | $120.64/120.64 |
| Materials | Program printing | 100 | $2/200 | $0/0 |
|  | Command strips for posters – bulk pack | 2 | $30/60 | $27.76/55.52 |
| Poster Awards | Gift cards | 2 | $100/200 | $105.95/211.90 |
| **Total** |  |  | **$2,110.00** | **$1,830.26** |
