# Supplementary material for: Building a Local Research Symposium: The Crossroads of Scholarship, Education, and Faculty Development
Source: MedEdPORTAL. 2020 Dec 24;16:11048. doi: 10.15766/mep_2374-8265.11048 (PMC7780738; doi:10.15766/mep_2374-8265.11048)
Supplement: Supplementary file 1 — Needs Assessment.docxSample Symposium Agenda.docxSymposium Planning Checklist.docxAbstract Submission Form.docxAbstract Quality Scoring Rubric.docxCorrespondence With Abstract Authors.docxPoster Session Moderator Instructions.docxPoster Session Moderator Scoring Sheet.docxSample Budget.docxSample Symposium Session Evaluation Forms.docx [file mep_2374-8265.11048-s001.zip › J. Sample Symposium Session Evaluation Forms.docx]

**Appendix J**

**Sample Symposium Session Evaluation Forms**

The following pages present sample individual session evaluations to be distributed during the appropriate sessions.

**Workshop Evaluation**

**Title**

**Facilitators: _____________**

Objectives:

1. Workshop objective #1
2. Workshop objective #2
3. Workshop objective #3

Strongly Strongly

disagree Disagree Neutral Agree agree

Workshop met objectives 1 2 3 4 5

[Presenter #1 name] was an effective presenter 1 2 3 4 5

[Presenter #2 name] was an effective presenter 1 2 3 4 5

Handouts were helpful to facilitate learning 1 2 3 4 5

Didactics were clear and informative 1 2 3 4 5

Small group exercises were beneficial 1 2 3 4 5

I will apply what I learned during the session 1 2 3 4 5

What did you learn today that you plan to apply to your scholarly work?

What is still unclear?

What was the most valuable aspect of this session?

How can we improve this session?

Should this topic be addressed at future symposia? What other topics would you like to see covered?

**Oral Presentations Session Evaluation**

**“Presentation #1 title”**

Strongly Strongly

disagree Disagree Neutral Agree agree

Presentation was well delivered 1 2 3 4 5

Presentation was well organized 1 2 3 4 5

Comments:

**“Presentation #2 title”**

Strongly Strongly

disagree Disagree Neutral Agree agree

Presentation was well delivered 1 2 3 4 5

Presentation was well organized 1 2 3 4 5

Comments:

**“Presentation #3 title”**

Strongly Strongly

disagree Disagree Neutral Agree agree

Presentation was well delivered 1 2 3 4 5

Presentation was well organized 1 2 3 4 5

Comments:

**“Presentation #4 title”**

Strongly Strongly

disagree Disagree Neutral Agree agree

Presentation was well delivered 1 2 3 4 5

Presentation was well organized 1 2 3 4 5

Comments:

What did you learn at this session that you plan to apply to your clinical or scholarly work?

What was the most valuable aspect of this session?

How can we improve this session?

**Oral Presentations Session Evaluation: “2 minutes, 2 slides, 2 questions: Clinical Case Symposium”**

**Please evaluate this session overall:**

Strongly Strongly

disagree Disagree Neutral Agree agree

Case presentations were well delivered 1 2 3 4 5

Key learning points were well described 1 2 3 4 5

This session should be held again next year 1 2 3 4 5

Comments:

What did you learn at this session that you plan to apply to your clinical or scholarly work?

What was the most valuable aspect of this session?

How can we improve this session?

**Poster Session Evaluation**

**I was a poster presenter YES NO**

**I participated in professor walk rounds YES NO**

**I was a facilitator/”professor” for walk rounds YES NO**

**I visited posters during the session YES NO**

Strongly Strongly

disagree Disagree Neutral Agree agree

The poster session was well organized 1 2 3 4 5

The professor walk rounds were constructive 1 2 3 4 5

I will apply what I learned during the session 1 2 3 4 5

I had enough time to visit posters that interested me 1 2 3 4 5

Posters covered a diverse array of topics 1 2 3 4 5

Overall poster quality was excellent 1 2 3 4 5

What did you learn today that you plan to apply to your clinical or scholarly work?

What is still unclear?

What was the most valuable aspect of this session?

How can we improve this session?
