# Supplementary material for: Building a Local Research Symposium: The Crossroads of Scholarship, Education, and Faculty Development
Source: MedEdPORTAL. 2020 Dec 24;16:11048. doi: 10.15766/mep_2374-8265.11048 (PMC7780738; doi:10.15766/mep_2374-8265.11048)
Supplement: Supplementary file 1 — Needs Assessment.docxSample Symposium Agenda.docxSymposium Planning Checklist.docxAbstract Submission Form.docxAbstract Quality Scoring Rubric.docxCorrespondence With Abstract Authors.docxPoster Session Moderator Instructions.docxPoster Session Moderator Scoring Sheet.docxSample Budget.docxSample Symposium Session Evaluation Forms.docx [file mep_2374-8265.11048-s001.zip › G. Poster Session Moderator Instructions.docx]

**Appendix G**

**Poster session moderator instructions and scoring rubric**

**Task list: Professor Walk Rounds/Moderated Poster Session**

1. Abstract scoring to determine abstracts for poster presentation (at least one month prior to symposium day).
2. Formal email invitation to poster presenters with poster instructions (at least one month prior to symposium day so the presenters get time to prepare and print the poster).
3. Numbering of the abstracts/posters (at least two weeks prior to the symposium day).
4. Formal email invitation to professors/moderators with assigned abstracts/posters and instructions (at least two weeks prior to the symposium day).
5. Put up the posters in the symposium venue as per designated poster numbers (at least one day prior to the symposium day).
6. Huddle with professors/moderators to go over the instructions of the professor walk rounds and distribute assigned abstracts and scoring sheet (on the day of the symposium).
7. Volunteers to keep a track of time (5 min for each poster presentation and 5 min for Q&A of each poster).
8. Collect feedback from poster session participants.
9. Collect scoring sheets from professors/moderators and decide “Best faculty poster” and “Best trainee poster.”
10. “Best faculty poster” award and “Best trainee poster” award declaration and distribution.

Select at least two independent moderators for each group of posters (for example Dr. XYZ and Dr. ABC for Posters 1 to 6 and Dr. UVW and Dr. DEF for posters 7-12), ask each of the moderators to score the abstract independently. At the end, the symposium committee should use average score for each poster for deciding the best poster award.

1 Mo Prior to Symposium

Abstract Scoring

Email invite to presenters with poster instructions

2 Wk. prior to Symposium

Abstract/Poster numbering

Email inite to moderators with assigned abstracts/poster and instructions

24 Hr. prior to symposium

Place the posters per assigned numbers

Print and group the abstracts and print scoring sheets for poster moderators

On the day of symposium

Huddle with moderators

Professor walk rounds

Scoring of posters and deciding best poster award

**Sample email invitation to poster presenter with poster Instructions (fill in bracketed information depending on symposium schedule) –**

Dear Dr. XYZ,

This year we have planned moderated poster session called “professor walk rounds” during our upcoming research symposium. It is an opportunity for our researchers like you to present your research topic at your poster. You will present your research topic over 5 minutes followed by Q&A for 5 minutes. The poster moderator/s (professor/s) will provide valuable input on the quality of your research study or case report, the quality of your presentation and the quality of your poster. They will also score your presentation and poster and review if it qualifies for the best poster award. This year we plan to select “The Best Faculty Poster Award” and “The Best Trainee Poster Award.” You are encouraged to join the professor/s on the walk rounds while they are reviewing other posters in order to create a collaborative environment of learning and networking as the professor/s move from one poster to another.

In order to participate in “professor walk rounds” and qualify for the best poster award, you are expected to hang up your poster at the symposium venue by **[date/time]** and be available at your poster on **[date]** between **[times]** to present your poster. Your abstract titled “**[title]**” has been designated number **[#]** so please remember to place your poster at your numbered spot in the symposium venue. Our research center associates will post abstract numbers, provide poster strips and assist you with hanging your posters in the symposium venue on **[date]**. Please do not bring your own pins or use push pins or tape to mount your posters as this will damage the walls.

The over-arching goals of the professor walk rounds at the symposium are to provide you with an ability to present your research work, get input from the experts, learn, network and have a lots of fun! So, please join us at **[time]** on **[date]** and share your work with all!

Research Symposium Committee

**Sample email invitation to professor/moderator for Professor Walk Round –**

Dear Sir or Madam: During our annual research symposium we have planned Professor Walk Round/Moderated Poster Session. It is an opportunity for our researchers to present their research topic at their respective posters. Each poster presenter will be asked to present his/her research topic over 3-5 minutes followed by Q&A for 3-5 minutes (details will be sent to you at least 2 weeks prior to the event).

Objectives of the “professor walk rounds”

1. Provide an opportunity to the researcher present his/her research topic at the poster
2. Provide an opportunity to the professor address key questions related to the research topic
3. Provide an opportunity to the professor address key components of quality of the presentation
4. Create a collaborative environment of learning and networking as the participants walk with the professor as a group from one poster to another

We would like to invite you as one of our experts to moderate our “professor walk rounds.” We will appreciate your valuable time, expertise and contribution. Once we have finalized the list of the abstracts for the symposium, we will assign you your abstracts/posters for you to moderate and evaluate. We will do our best to assign you abstracts/posters, which are related/close to your area of expertise. We will send you your assigned abstracts, details of the walk rounds (time allotted per poster, details of scoring, etc.) at least 2 weeks prior to the event. Please block your calendar for the professor walk rounds between **[times]** in **[location]** on **[date]**. This year again, we plan to select at least 1 faculty and 1 trainee as our poster session winners based on the scores gathered from the moderators at the professor walk rounds.

Please let us know your availability to moderate the professor walk rounds by **[deadline]**. We will appreciate your support. We look forward to a successful event. Thank you.

Research Symposium Committee
